# Impact of a Self-Autonomous Evaluation Station and Personalized Training Algorithm on Quality of Life and Physical Capacities in Sedentary Adults: Randomized Controlled Trial

**DOI:** 10.2196/45461

**Published:** 2024-10-04

**Authors:** Yann Le Mat, Corentin Casali, Franck Le Mat, Léonard Féasson, Clément Foschia, Mathias Géry, Jérémy Rossi, Guillaume Y Millet

**Affiliations:** 1 Université Jean Monnet Saint-Etienne Lyon 1, Université Savoie Mont-Blanc Laboratoire Interuniversitaire de Biologie de la Motricité, F-42023 Saint Etienne France; 2 Centre Hospitalier Universitaire (CHU) Saint-Etienne Service de Physiologie Clinique et de l’Exercice, Laboratoire Interuniversitaire de Biologie de la Motricité Saint Etienne France; 3 Centre Hospitalier Universitaire (CHU) Saint Etienne Centre Référent Maladies Neuromusculaires Rares - European Reference Networks (ERN EuroNmD) Saint Etienne France; 4 Université Jean Monnet Saint-Etienne, Centre National de la Recherche Scientifique (CNRS), Institut d'Optique Graduate School, Laboratoire Hubert Curien, Unité Mixte de Recherche (UMR) 5516, F-42023 Saint Etienne France; 5 Institut Universitaire de France (IUF) Paris France

**Keywords:** physical activity, sedentary behavior, quality of life, mobile health, health-related interventions, mobile app, mobile phone

## Abstract

**Background:**

Physical inactivity is a major risk factor for noncommunicable diseases and a leading cause of premature death. The World Health Organization (WHO) recommends at least 150 minutes of moderate intensity physical activity (PA) weekly, regardless of age, gender, or personal habits. However, in both sports performance and clinical settings, personalized training (PT) regimens have shown superior efficacy over general guidelines.

**Objective:**

We hypothesized that an automatic PT program, informed by initial physical evaluations, would increase overall quality of life, quality of sleep, and physical capabilities and reduce fatigue and depression compared with adherence to WHO recommendations.

**Methods:**

This 5-month, randomized, single-blinded controlled trial involved 112 sedentary or minimally active participants, divided randomly into PT and free training (FT) groups. Physical capabilities and subjective measures such as quality of life, sleep, depression, and fatigue were evaluated for both groups. After 1 month, both groups were asked to perform 150 minutes of PA per week for 4 months; the PT group could either follow a “virtual coach” on a mobile app to follow some personalized PA or do what they would like, while the FT group was to follow the general PA recommendations of the WHO.

**Results:**

We did not find any group×time interaction for PA duration or intensity, physical qualities, and subjective measures. However, considering both groups together, there was a significant pretest and posttest time effect for duration of PA (18.2 vs 24.5 min/d of PA; *P*<.001), intensity (2.36 vs 3.11; *P*<.001), and workload (46.8 vs 80.5; *P*<.001). Almost all physical qualities were increased pretest and posttest (ie, estimated VO_2_max 26.8 vs 29 mL min^–1^ kg^–1^; *P*<.001; flexibility 25.9 vs 26.9 cm; *P*=.049; lower limb isometric forces 328 vs 347 N m; *P*=.002; reaction time 0.680 vs 0.633 s; *P*<.001; power output on cyclo-ergometer 7.63 vs 7.82 W; *P*<.003; and balance for the left and right leg 215 vs 163 mm^2^; *P*<.003 and 186 vs 162 mm^2^; *P*=.048, respectively). Finally, still considering the PT and FT groups together, there were significant pretest to posttest improvements in the mental component of quality of life using the 12-item Short Form Health Survey (41.9 vs 46.0; *P*<.006), well-being using the Warwick-Edinburgh Mental Well-Being Scale (48.3 vs 51.7; *P*<.002), depression using the Center for Epidemiologic Studies Depression Scale (15.5 vs 11.5; *P*=.02), and fatigue using the Functional Assessment of Chronic Illness Therapy–Fatigue (37.1 vs 39.5; *P*=.048).

**Conclusions:**

The individualized training was not more effective than the general recommendations. A slight increase in PA (from 18 to 24 min/d) in sedentary or poorly active people is enough for a significant increase in physical capabilities and a significant improvement in quality of life, well-being, depression, and fatigue.

**Trial Registration:**

ClinicalTrials.gov NCT04998266; https://clinicaltrials.gov/study/NCT04998266

## Introduction

### Physical Activity and Health

A low level of physical activity (PA) and a predominantly sedentary lifestyle are major risk factors for noncommunicable diseases such as cardiovascular disease, chronic obstructive pulmonary disease, cancer, and type 2 diabetes [1-3]. Noncommunicable diseases are the foremost causes of death globally [4]. According to the World Health Organization (WHO), quoting the work of Lee at al [5], physical inactivity is the cause of 5% of coronary heart disease, 7% of type 2 diabetes, 9% of breast cancers, and 10% of colon cancers. Scientific data now clearly demonstrate the favorable effects of PA in the prevention of chronic pathologies [6]. In addition to its effects on physical health, PA is also recognized for its mental health benefits, with several studies showing that engaging in regular activity can reduce symptoms of depression and anxiety, which can also improve mood and help manage stress [7-9]. It has also been shown that PA can improve overall sleep quality [10]. The consensus is that regular PA markedly enhances quality of life [11,12].

The beneficial effects of PA on health and the harmful effects of a sedentary lifestyle have led to the development of recommendations to guide the population in acquiring an active and healthy lifestyle. For people aged 18 to 65 years, the recommendations from the WHO and from the American College of Sports Medicine are to perform moderate PA, at least 30 minutes a day, 5 times a week, for a total of 150 to 300 minutes per week, avoiding 2 consecutive days without PA [13]. However, to date, the threshold of a sedentary lifestyle harmful to health has not yet been clearly defined. According to Chau et al [14], each additional hour spent in a seated position increases the risk of mortality by 2% between 3 and 7 hours of sedentary time and by 5% beyond 7 hours of sedentary time. In addition, PA improves the work performance of employees [15], and some recent surveys show that 53% of employees would welcome a sports offer from their company and that the employees could increase their exercise time by as much as 34% when given a motivating and individualized program [16].

Despite all positive impacts of PA on health, there are many barriers to actually exercise, which explains why the level of PA in the general population remains low. Known barriers to PA across a range of settings include lack of time, fatigue, lack of knowledge, poor self-efficacy, lack of social support, and self-motivation [17].

### Technology and Physical Activities

Information and communication technologies appear to be a promising tool for providing recommendations, providing personalized follow-up and real-time feedback and, in short, improving compliance to PA requirements. Recent reviews and meta-analyses have found web- and smartphone-based interventions to be effective in increasing PA in the general population. Some studies have investigated whether new technologies could have a positive impact on the amount of PA. In their systematic review, Muntaner et al [18] show that half of the articles (6/12, 50%) analyzed show an increase in the level of PA through the use of apps on mobile phones. Davies et al [19] showed in a meta-analysis that interventions delivered on the internet can increase the amount of PA both in sedentary people and in patients. More recently, the systematic review of Buckingham et al [20] shows that the use of “mobile health” is a good alternative for promoting PA at the workplace. The COVID-19 pandemic has further underscored the significance of digital interventions in public health [21].

### Personalized Training

General PA recommendations are the same for everyone, regardless of age (at least between 18 and 65 years), gender, physical capacities, type of professional activity, body composition, and so on.

Current general recommendations for PA emphasize the importance of engaging in at least 150 to 300 minutes of moderate intensity aerobic activity per week or 75 to 150 minutes of vigorous intensity aerobic activity. In addition, it is recommended to include muscle-strengthening activities on ≥2 days a week. These guidelines apply universally, regardless of age (specifically between 18 and 65 years), gender, physical capacities, type of professional activity, body composition, and so on. This consensus is supported by numerous studies, including the study by Bull et al [22], which provides comprehensive evidence on the health benefits of regular PA across diverse populations.

In the field of sports performance (eg, see the study by Weineck et al [23]), and in the clinical field, it is recognized that individualized training is more effective than general recommendations. For example, it was shown that when PA is adapted to the symptoms, the reduction in the level of perceived fatigue was greater in patients with cancer [24] or multiple sclerosis [25]. Brown et al [24] suggested that exercise interventions need to be tailored based on health outcomes. Therefore, the recommendations of PA adapted and personalized to the deficits of the patients should lead to better quality of life than a classic PA intervention. We hypothesized that a tailored intervention should also be efficient in sedentary or poorly active people.

### Profiling People

In order to tailor the training intervention to the physical capacities of the individuals, it is fundamental to appropriately evaluate them before the program [26]. For instance, the use of an objective method using an ergometer to assess aerobic capacity, rather than the use of field testing (ie, 6-minute walk test), has been proposed by Genin et al [27]. We decided to also use objective measures of participants’ main physical capacities and capabilities to get the most accurate evaluation of their fitness level, identifying strengths and weaknesses with respect to gender and age.

### Objectives

We hypothesized that an automatic personalized training (PT) program based on some initial physical evaluations would increase the overall quality of life, physical capabilities, and quality of sleep, as well as reduce fatigue and depression. The main objective of this study was to evaluate the effectiveness of our personalized intervention compared with applying the standard WHO recommendations.

## Methods

### Ethical Considerations

This study was conducted in accordance with the Declaration of Helsinki and approved by a national ethics committee (CPP Tours Ouest 1; approval number 2021T2-19 HPS). Our study involved human subjects and adhered strictly to ethical standards for research. All participants provided written informed consent before their involvement in the study. They were fully informed about the study’s procedures, its purpose, the voluntary nature of their participation, and their right to withdraw at any time without any consequence. To protect participants’ privacy and confidentiality, all personal data were anonymized. Access to these data was limited to researchers directly involved in the study. Instead of financial compensation, participants were allowed to keep the mobile app used during the study for ongoing personal use, free of charge. This app assists with PA, contributing to long-term health benefits for the participants. No individual participants are identifiable in any images or supplementary material associated with this manuscript.

### Study Design

This study was a 5-month, randomized, single-blinded controlled trial that entailed a 1-month observation period and a 4-month period of prescribed PA. Participants were randomized 1:1 to either the intervention group or the control group.

The design of our study evolved from comparing the 2 groups to a pretest-posttest comparison for a single group (both groups pooled together).

### Participants and Recruitment

Enrollment and follow-ups were conducted between September 2021 and February 2022. Participants were recruited through flyers and emails sent to employees of Jean Monnet University and Conseil Général de La Loire (Saint-Étienne, France) and via articles in local newspapers. The inclusion criteria were that the participants must be aged between 18 and 65 years, male or female, sedentary people (maximum 1 session of 1 hour of PA per week) sitting >3 hours a day, and having smartphone or tablet access to the internet.

This study specifically excluded some populations due to safety considerations and relevance to the research objectives. Exclusion criteria included individuals with degenerative diseases, as the recommended PAs might negatively impact their condition. Pregnant women were also excluded to avoid any potential risks associated with exercise during pregnancy. Participants with cardiovascular issues were not included, considering the associated risks of PA under these conditions. In addition, participants who were unable to understand the study’s procedures or provide informed consent were excluded to ensure ethical standards and the validity of the outcomes. Furthermore, all willing participants were orally questioned to ascertain their level of sedentariness and inactivity, specifically if they remained seated for >3 hours per day and if they engaged in <1 hour of PA per week. This information was crucial to identify individuals who met our study’s criteria.

Eligible participants underwent a medical examination with one of the doctors of the laboratory who, after checking that they could safely follow the study protocol, included them in the trial after the participants provided informed consent. Immediately after this medical examination, randomization of the participants was performed using a centralized, secured management system, REDCap (Research Electronic Data Capture; Vanderbilt University). At registration, participants were asked to download a custom-developed mobile app (Ionic and Angular JS application framework, available on App Store [Apple] and Play Store [Google]) and to register an account. Initially, participants were required to complete 5 questionnaires through the mobile app that aimed at gathering information on various subjective measures.

### Measurements and Follow-Up

The data collected during the study and follow-up were recorded using the mobile app and saved on the university server. Each month, exports were done to fill a centralized, secured management system, REDCap, in accordance with ethical committee recommendations.

**Figure 1:**
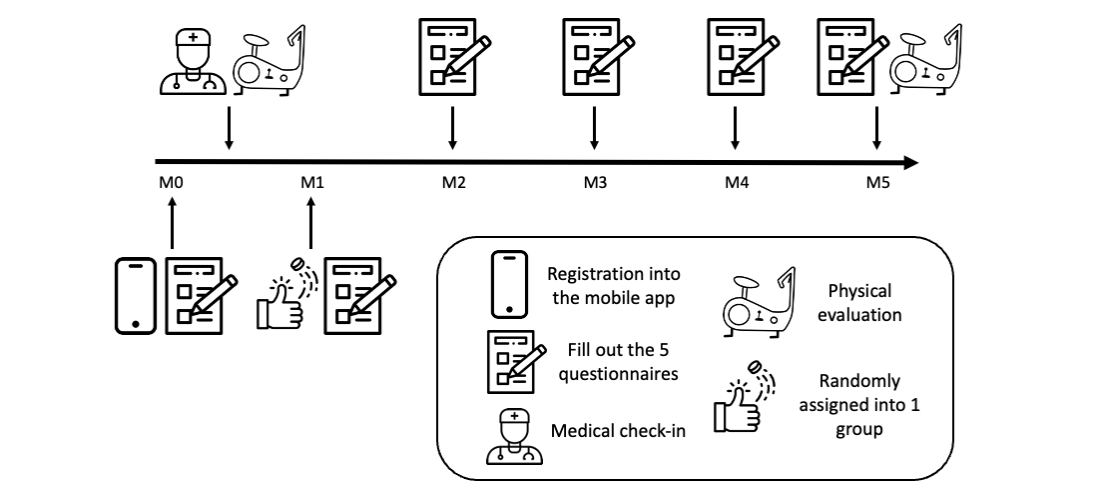
Detailed study flowchart and timelines for sedentary adult participants: this figure illustrates the comprehensive process and timelines of our randomized controlled trial. Starting with enrollment (M0), sedentary adult participants were recruited, and they underwent a medical examination to ensure eligibility. Following registration in the custom-developed mobile app, participants were asked to complete 5 questionnaires to capture baseline health data. After a physical evaluation (M0), participants were randomly assigned into groups for the intervention. Each subsequent month (M1-M5) involved the same 5 questionnaires. A final physical evaluation was done at the end of the study period (M5). FT: free training; PT: personalized training.

Each participant performed a medical check-in with a physician who gave a complete explanation of the study. Then, the participants performed the physical assessment with the main instructor of the experiment (Figure 1). Demographic variables including gender, date of birth, height, body mass, and percentage body fat using bioelectrical impedance (Tanita RD-545 HR; Tanita Corporation) were also included.

### Objective Physical Capacities Tests (Station)

**Figure 2:**
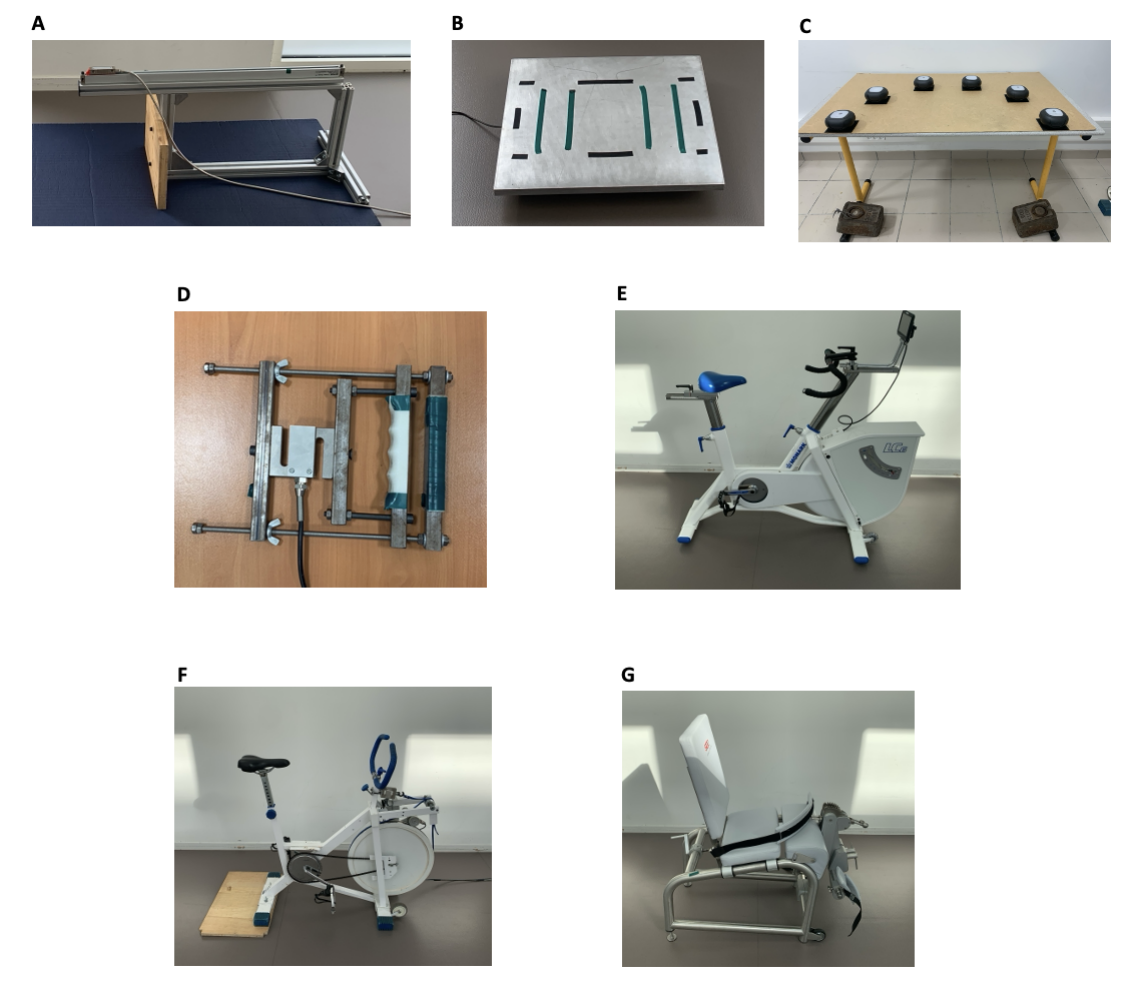
Assessment equipment for measuring physical fitness in sedentary adults: this figure presents the 7 tools used to evaluate the physical fitness of sedentary adults. These include (A) a custom sit and reach device for flexibility measurement, (B) a stabilometry platform to assess balance, (C) a reaction time–testing setup, (D) a custom dynamometer for grip strength measurement, (E) a cyclo-ergometer used for indirect VO2max estimation, (F) a friction-load cycle ergometer for the force-velocity test, and (G) a dynamometric chair for assessing the maximal strength of knee extensors.

**Figure 3:**
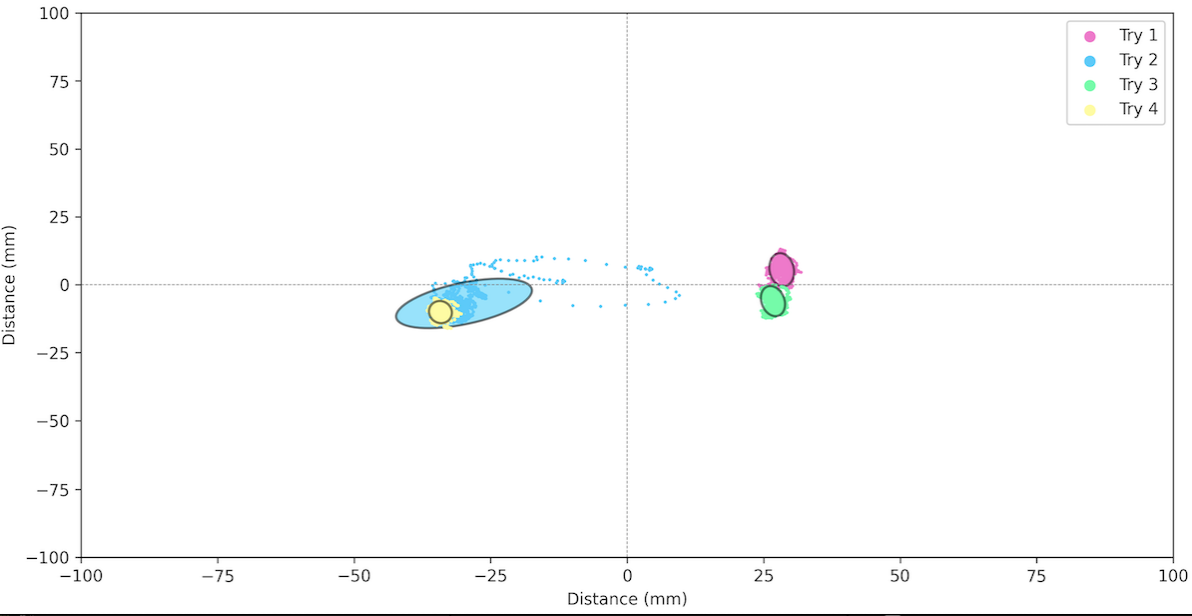
Example of the ellipse area for the balance test.

**Figure 4:**
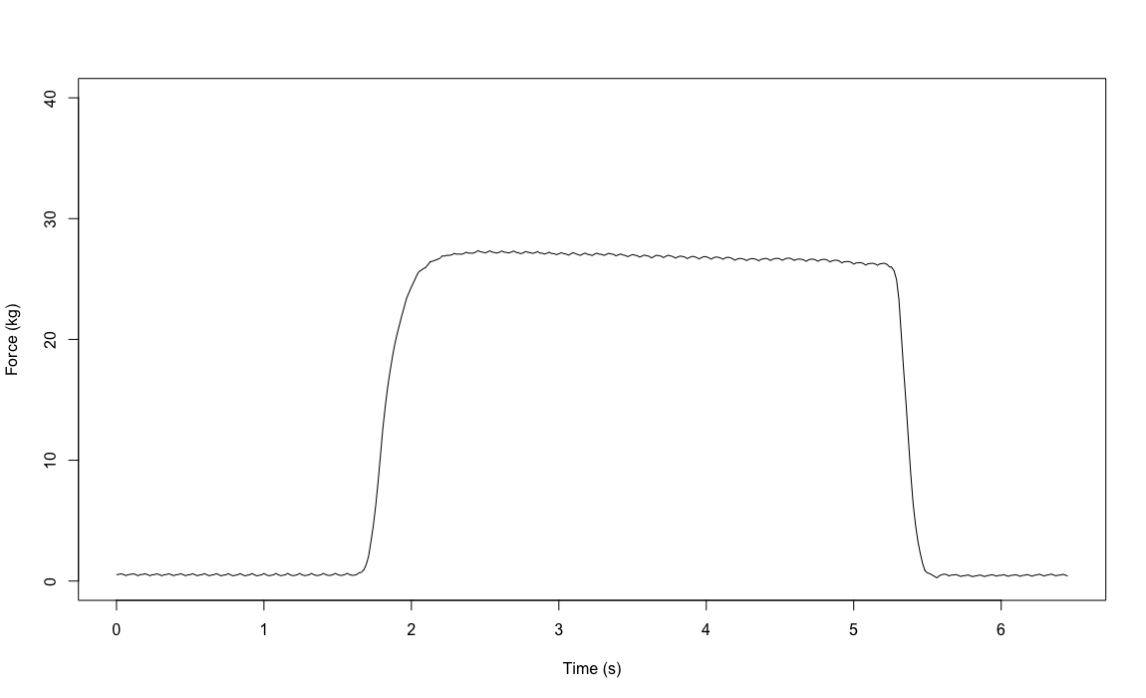
Example of the signal acquired for the handgrip test.

**Figure 5:**
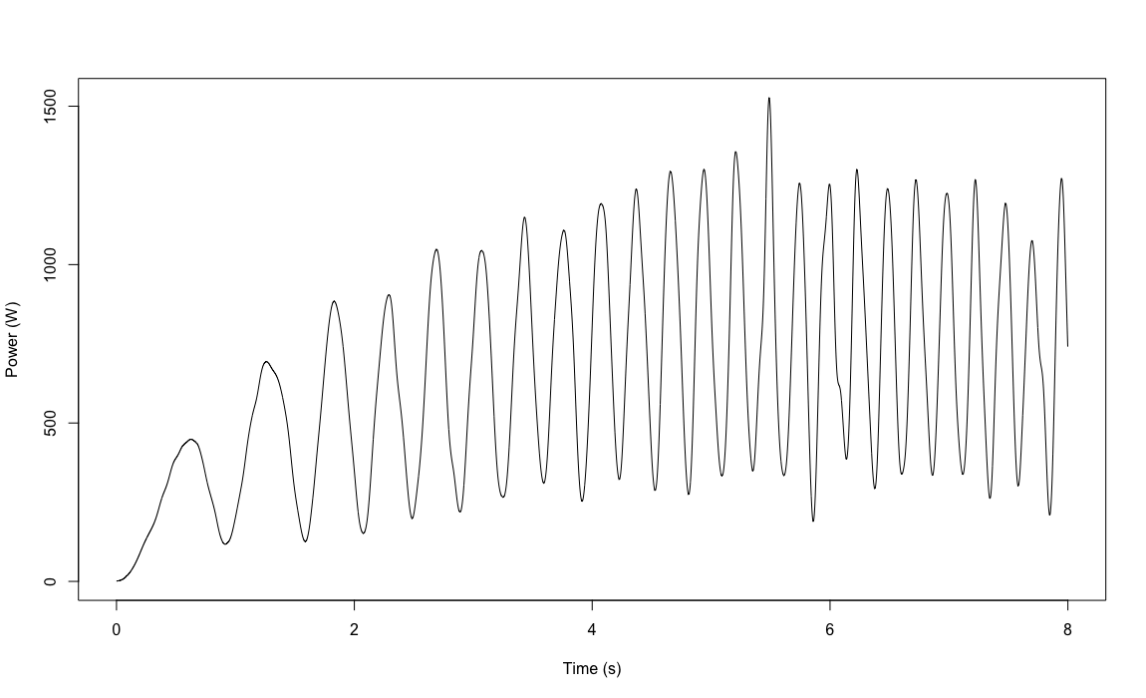
Example of the signal of the force-velocity test.

**Figure 6:**
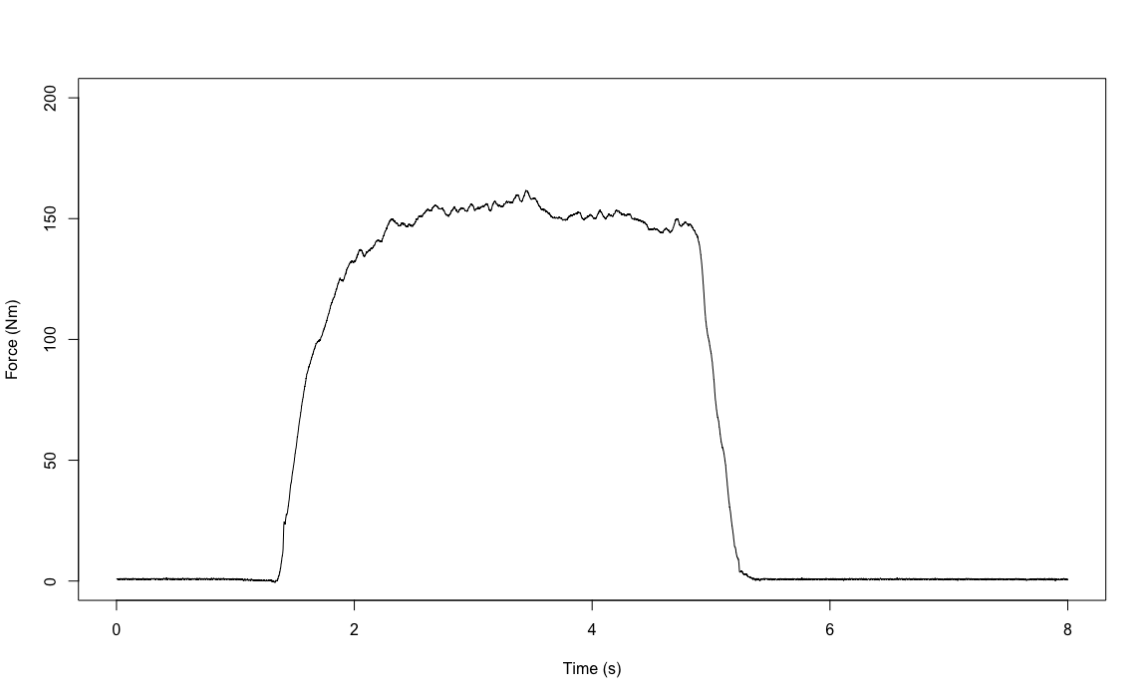
Example of the signal acquired in the leg extension test.

Physical fitness was evaluated in both groups based on 7 tests (Figure 2). First, flexibility was measured with a custom, instrumented (Figure 2A) Sit and Reach Test [28] using a linear encoder to measure the distance (Linear Encoder 1100mm; Phidgets Inc). Participants performed 2 trials separated by 30 seconds of rest. Second, balance was assessed with a single leg balance test performed on both legs. The test involves staying as immobile as possible on 1 leg on a platform for 15 seconds while fixing a fixed point on the wall. Measurements were obtained using a customized stabilometry platform (Figure 2B, data were acquired at 62 Hz and were then filtered with a fourth-order low-pass Butterworth filter at 20 Hz). The other leg was free in the air; the only rule was to not touch the other leg (ie, the one standing on the custom platform). Participants performed 2 trials by leg with 30 seconds of rest between tests. The 95% ellipse area (in mm^2^) was computed; it represents the area covered in the mediolateral and anteroposterior direction (an example of the ellipse area is available in Figure 3). It is considered to be an index of the overall postural performance; the smaller the surface, the better the performance [29]. Third, reaction time was evaluated using a custom test (Figure 2C) inspired from the study by Zwierko et al [30]. The test consisted of 6 units (ROXs Pro) placed in a semicircle on the table. When these units lit up randomly one by one, the goal was to touch the lighted unit as fast as possible to turn it off. Each light was set to light up for a time between 0.1 and 3 seconds to avoid the participant getting used to a rhythm. A total of 22 touches were performed. The reaction time was computed for each correct touch. Participants performed 2 trials with 1 minute of rest between trials. Fourth, grip strength was measured on both hands using a custom dynamometer (Figure 2D, data were acquired at 62 Hz. An example of the data acquired is available in Figure 4) equipped of a gauge strain and designed to have the same size (ie, 4.8 cm) as the Jamar (Sammons Preston Rolyan). Participants performed 2 maximal voluntary contractions per side with 30 seconds of rest between trials and were vigorously encouraged to squeeze as hard as possible during each trial. Fifth, VO_2_max was indirectly evaluated using the test of Astrand [31], which is a submaximal effort of 6 minutes on a cyclo-ergometer (Figure 2E; Monark LC6 novo). The estimated VO_2_max was calculated using a nomogram that links the average heart rate and the average power output between minute 4 and 6. The nomogram is calibrated to gender and age. Sixth, a force-velocity test was performed on a friction-load cycle ergometer (Figure 2F; Monark) to measure maximal power. All features of the ergometer have been detailed in previous studies [32,33]. Briefly, the signal was acquired at 1000 Hz, and the data were then filtered with a fourth-order low-pass Butterworth filter at 20 Hz (an example of the signal is in Figure 5). Participants performed 2 sprints lasting 8 seconds maximum, separated by a 2-minute rest period, with friction loads of 0.3 N.kg^–1^ and 0.4 N.kg^–1^ body mass for women and 0.4 N.kg^–1^ and 0.7 N.kg^–1^ for men, respectively. They were vigorously encouraged to pedal as fast as possible during the entire sprint. Seventh, the knee extensors’ maximal strength was assessed on a dynamometric chair (Figure 2G; ARS dynamometry, S2P Ltd). Data were acquired at 1000 Hz and filtered with a smoothing window of 0.005 seconds (an example of the signal is available in Figure 6). Participants were seated in an upright position on the chair with both the right knee and hips at 90° of flexion, with the chest and hips securely strapped. Participants performed two 4- to 5-second maximal voluntary contractions with 1 minute of rest between trials. They were vigorously encouraged to exert force as intensely as possible during the task.

### Subjective Measurements (Questionnaires)

Quality of life was assessed using the 12-item Short Form Health Survey (SF-12; version 2). The SF-12 assesses functional limitations via 12 items. It consists of 2 subscales measuring physical health (physical component subscale; PCS) and mental health (mental component subscale; MCS). It rates the presence and severity of different impairments over the past 4 weeks. The following subjective measures were also assessed: fatigue using Functional Assessment of Chronic Illness Therapy–Fatigue (FACIT-F), quality of sleep using Insomnia Severity Index (ISI), well-being using the Warwick-Edinburgh Mental Well-Being Scale (WEMWBS), and depression using the Center for Epidemiologic Studies Depression Scale (CES-D). Each questionnaire was to be completed every month for 5 months (Figure 1).

### PT and Free Training Groups

**Figure 7:**
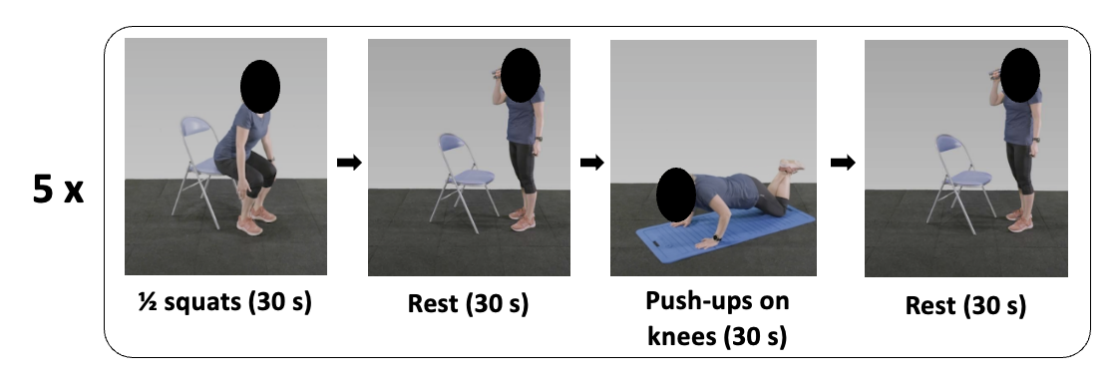
Sample exercise sequence from the mobile app for home-based physical activity: a 10-minute home exercise sequence used in our study for enhancing physical capacity in sedentary adults. This example includes repeated cycles of half squats for 30 seconds, followed by a rest period of 30 seconds, push-ups on knees for 30 seconds, and another rest interval of 30 seconds, to be completed 5 times. The sequence is designed to be performed with minimal equipment, requiring only a chair and a mat, facilitating accessibility for participants.

The intervention group (PT) beneficiated, for 4 months, from a custom mobile app designed to automatically recommend personalized PA (this feature was called the “virtual coach”), aiming to help participants achieve the recommended levels of PA. An algorithm was developed to recommend PA sessions, considering the participant’s profile (based on his or her physical capabilities evaluated beforehand). The personalized PA sessions are based on predefined sequences of 10 minutes from a database of >500 sequences, using approximately 100 different exercises (see an example in Figure 7).

**Figure 8:**
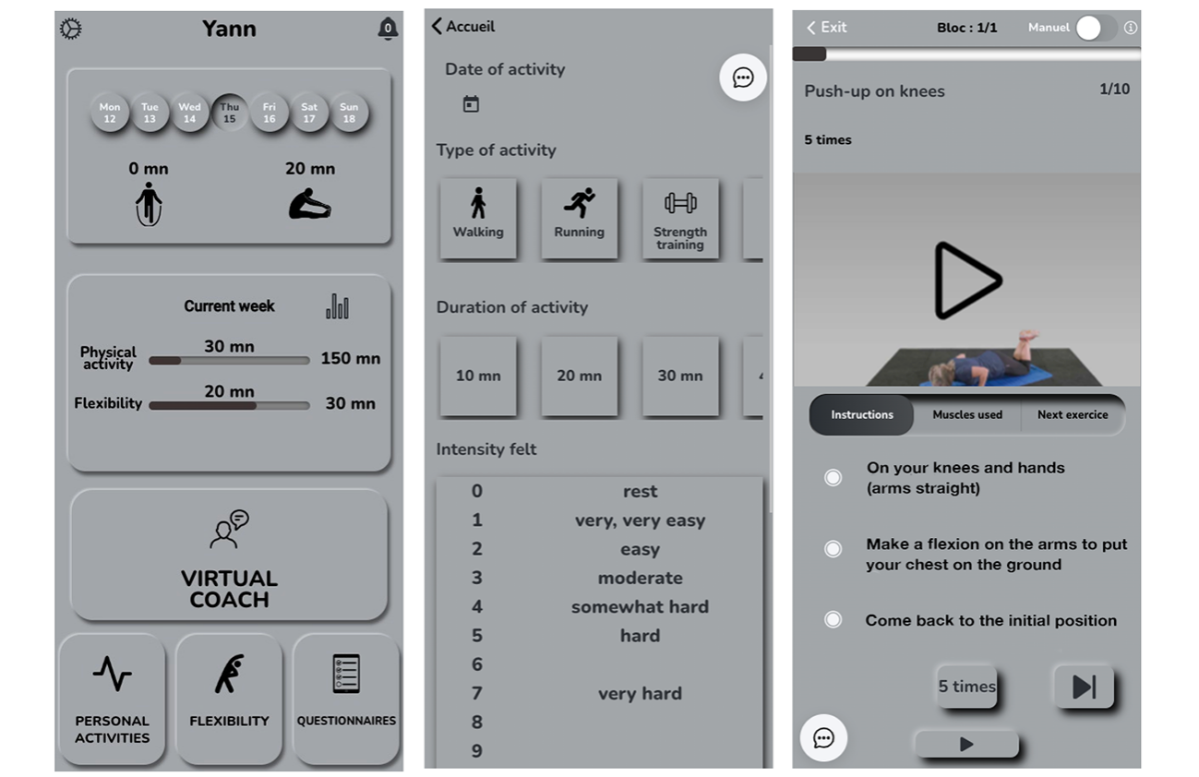
This image showcases the mobile app developed for the study, displaying the home screen with gauges for tracking time spent on physical activity and stretching (left), a self-report section for physical activity input (middle), and a personalized virtual coach screen providing detailed exercise instructions (right). Screenshots are translated into English for clarity in this paper, and the color scheme has been adjusted for enhanced legibility.

**Figure 9:**
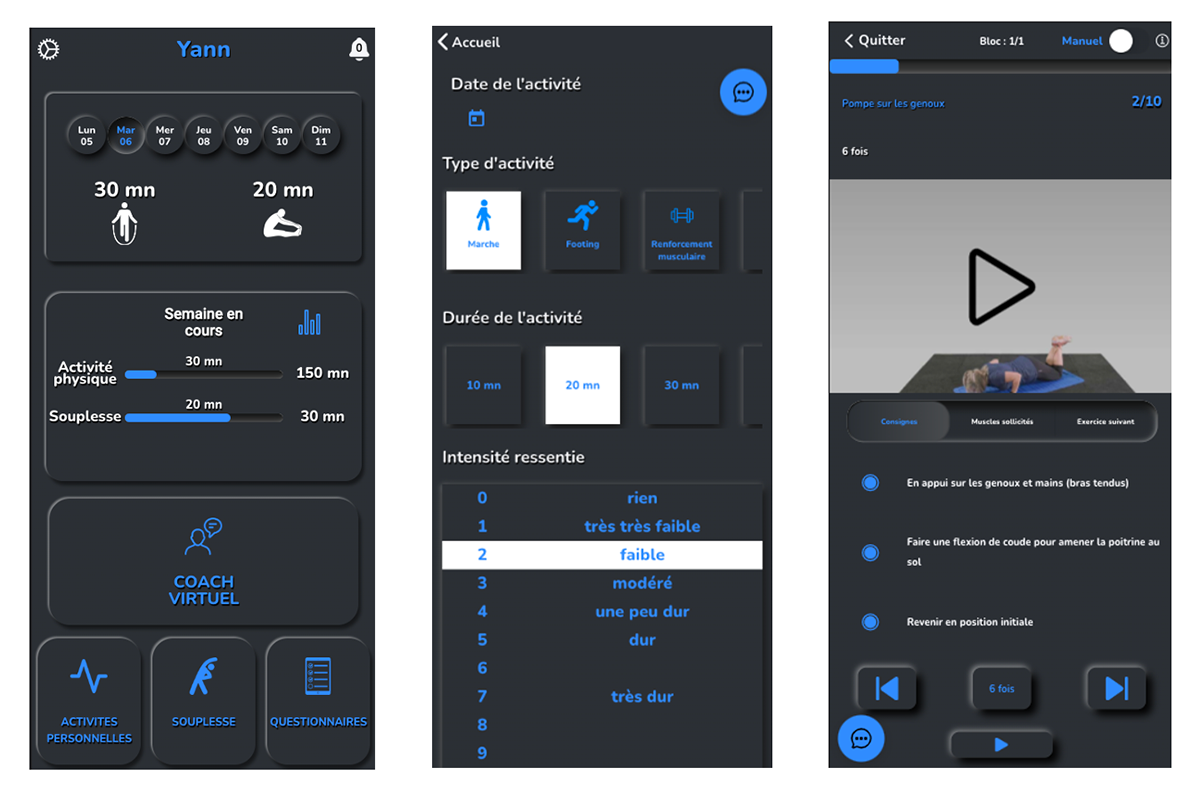
Original screenshots of the mobile app.

Each sequence was classified according to its nature (aerobic, strength, or balance) and difficulty. The algorithm selected the appropriate sequence based on the participant’s physical capacities; the available sports equipment (such as dumbbells, Swiss ball, bands, bike ergometer, and jump rope); location (indoor or outdoor); and the available or desired duration (multiples of 10 minutes, from 10 minutes to 60 minutes). Each sequence was composed of 1 to 3 different exercises. Each exercise was set either as a period of time (eg, 30 s) or as a number of repetitions. Exercises were separated by periods of rest. For each exercise, the mobile app displayed a short explanation and a video showing how to perform the exercise properly (Figures 8 and 9). After each sequence of 10 minutes, the rating of perceived effort (RPE) was to be reported using the Borg Scale [34]. RPE was considered by the algorithm for the next session; an RPE ≥2 led to an increase in the difficulty of the following session, and to the opposite, an intensity ≥5 selected an easier next-session exercise. For further analysis, the workload of each session was calculated as the RPE (ie, intensity) multiplied by the duration. The home page showed the goal of 150 minutes of PA per week to follow the WHO’s recommendations. A gauge was displayed, and each time the participant was exercising, the gauge increased until she or he reached the goal (Figures 8 and 9). If the participant exercised more than the goal, the displayed value increased, but the gauge stayed full. In addition to the sessions recommended by PT, the participants were also allowed to add their own activities on the mobile app, for example, walking, cycling, swimming, rowing, fitness or gardening, and housework. These activities were considered only if the duration was at least 10 minutes (Figures 8 and 9). Finally, the participant could perform some stretching sessions that were not considered as PA. So, the PT group was allowed to perform whatever they wanted to achieve in the 150 minutes of PA each week; that is, they could follow their desired percentage of the PT.

Participants allocated to the control group (free training [FT]) received the same mobile app without the “virtual coach.” The 150 minutes gauge was displayed on the home page as was displayed for the intervention group, and the participants had to add all their PAs manually. To help them to reach their goal, the general guidelines (ie, the goal is to reach 150 minutes of PAs per week of aerobic exercises at moderate intensity and do some resistance training for at least 2 times a week) [35] of PA were written in the mobile app.

### Sample Size

On the basis of the hypothesis of a difference of 4.2 points (SD 5.8) [36,37] in the variation of the SF-12 score between the 2 types of training, with an α risk of 2.5 (Bonferroni correction, the score of SF-12 being composed of 2 subscores corresponding to the PCS and the MCS) and a power of 90%, we initially calculated a requirement of 48 participants per group. To accommodate an anticipated dropout rate of 20%, we adjusted the number to 58 participants per group, ensuring robustness against potential loss to follow-up.

The adjustment of the study (from comparing 2 groups with a pretest-posttest comparison for a single group) changes the statistical power calculation. For the adjusted design, focusing on a power of 90%, a mean difference of 4.2 (as per Jayadevappa et al [36]), an SD of 5.8, and an α of .025 (considering the Bonferroni correction for 2 components of the SF-12), the required sample size was 23 participants. The initial sample rate calculation largely fits with the new design, which makes the statistical analysis powerful enough.

### Data Analysis

In order to keep the same duration between pretest and posttest, we defined the posttest period as the last 30 days counting backward from the posttest date.

### Statistics

Statistical analysis was performed with R (R Foundation for Statistical Computing). Results are expressed as mean and SD. Normality of the data was assessed via Q-Q plot and the Shapiro-Wilk test. The presence of outliers was evaluated using the IQR method. Mixed model ANOVA was computed with group (FT vs PT) as the between-group factor and with time (pretest vs posttest) as the within-subject factor.

For each ANOVA conducted, the Mauchly test was used to assess sphericity for the dependent variables in within-group analyses. The Levene test was used to check the assumption of equality of variances for between-group analyses. The test was performed at each time frame, pretest and posttest. To check the assumptions of the homogeneity of variances of the between-subject factor (FT vs PT), the Box M test was used, with a significance threshold of α=.01.

Bonferroni corrections were only applied to pairwise comparisons to follow up a significant main effect for condition (ie, time effect). When there was no time×group interaction, the main group effect (FT vs PT) or time effect (pretest and posttest) was assessed using pairwise comparisons. Statistical significance was set at *P*<.05.

Finally, Pearson correlation coefficients have been computed between all variables (ie, pretest, posttest, and pretest-posttest variations of subjective measures; physical capabilities; and PAs) to check for correlation between any of these parameters.

## Results

### Participants

The recruitment was conducted from September 2021 to February 2022. A total of 112 participants were enrolled and randomly assigned to either FT (n=54, 48.2%) or PT (n=58, 51.8%). Participants’ characteristics are presented in Table 1. The mean age of the sample was 45.1 (SD 9.05) years, and 74.1% (83/112) of them were women.

**Table 1:** Demographic and physical characteristics of study participants.

		Personalized training group (n=58)	Free training group (n=54)	Total (n=112)
**Age (y)**				
	Mean (SD)	45.2 (9.02)	45.1 (9.16)	45.1 (9.05)
	Median (IQR)	47.0 (22.0-60.0)	46.0 (24.0-59.0)	46.0 (22.0-60.0)
**Gender, n (%)**				
	Women	45 (77.6)	38 (70.4)	83 (74.1)
	Men	13 (22.4)	16 (29.6)	29 (25.9)
**Body mass (kg)**				
	Mean (SD)	75.5 (16.6)	78.5 (18.3)	76.9 (17.4)
	Median (IQR)	73.1 (49.4-136.0)	74.7 (51.0-118)	74.6 (49.4-136.0)
**BMI (kg/m²)**				
	Mean (SD)	26.7 (4.5)	27.4 (5.6)	27.0 (5.1)
	Median (IQR)	26.2 (19.8-39.6)	26.3 (18.9-41.9)	26.2 (18.9-41.9)

We observed a dropout of 33.9% (38/112) during the study, meaning that 74 participants came back at the second physical evaluation (posttest).

Of the 74 participants who came back, 44 (59%) to 47 (64%) participants (depending on the questionnaires) filled out the questionnaires at posttest (<20 days before or after the second evaluation).

### Duration, Intensity, and Workload

**Figure 10:**
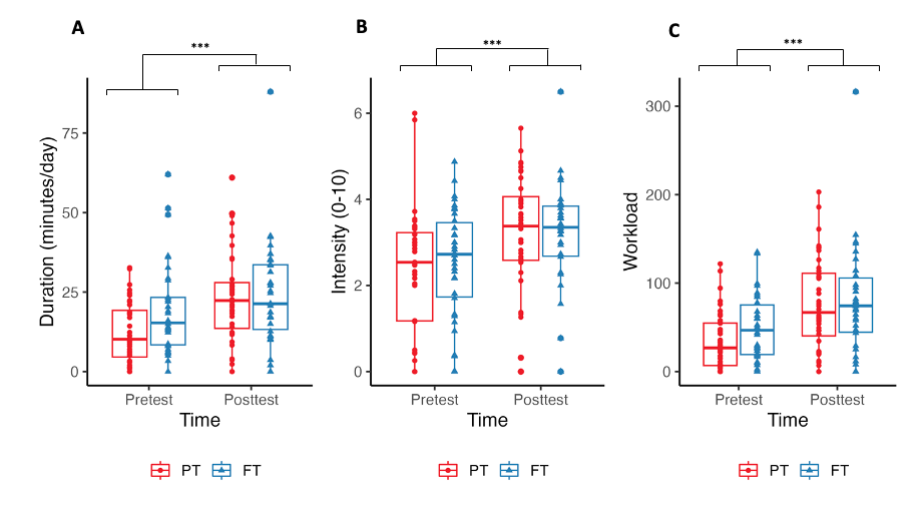
Comparison of exercise duration, intensity, and workload in personalized (PT) versus free training (FT) programs. This box plot illustrates the variations in exercise duration (min/d), perceived intensity (scale 0-10), and total workload before and after the intervention for both PT and FT groups. Statistical comparisons are shown for the 2 time points (pre- and postintervention time points) across the groups. Significant differences are indicated by asterisks.

There was no group×time interaction for PA duration (*P*=.055), intensity (*P*=.54), or workload (*P*=.21). However, the PT group was doing less PA than the FT group at pretest (mean 22.4, SD 16.9 vs mean 14.6, SD 11.7 min/d; *P*=.02; Cohen *d*=0.54) but not at posttest (Figure 10). The assignment to groups at pretest was done independently from prior PA levels. The PT group only used the PT a mean 0.85 (SD 1.76) minutes per day.

Daily average duration, intensity, and workload are presented in Table 2. There was a significant time effect for the average duration, intensity, and workload.

**Table 2:** Pre- and postintervention changes in physical activity characteristics.

		Pretest (n=74)	Posttest (n=74)	Change (%)	*P* value	Effect size	
**Duration (min/d)**							
	Mean (SD)	18.2 (14.8)	24.5 (15.3)	34.6	<.001	0.403 (small)	
	Median (range)	15.6 (0-77.7)	23.2 (0-88.0)	—^a^	—	—	
**Intensity (0-10)**							
	Mean (SD)	2.36 (1.32)	3.11 (1.32)	31.7	<.001	0.600 (moderate)	
	Median (range)	2.53 (0-6.00)	3.34 (0-5.65)	—	—	—	
**Workload**							
	Mean (SD)	46.8 (41.1)	80.5 (57.5)	72	<.001	0.647 (moderate)	
	Median (range)	35.3 (0-191)	75.0 (0-316)	—	—	—	

^a^Not applicable.

### Questionnaires

There was no time×group interaction for any questionnaire, but a time effect was found for the MCS of SF-12, Well-Being Scale (WEMWBS), CES-D, and FACIT-F (Table 3). For the FACIT-F, 40% (18/44) of the participants were considered as “fatigued” (score=34) at pretest versus 18% (8/44) at posttest. No time effect was found for the PCS of SF-12 or for the ISI.

**Table 3:** Comparative analysis of questionnaire outcomes before and after the intervention.

				Pretest (n=74)		Posttest (n=74)		Change (%)		*P* value		Effect size
**SF-12^a^**												
	**Mental component subscale (n=47)**											
		Mean (SD)	41.9 (7.07)		46.0 (9.99)		9.8		.006		0.415 (moderate)	
		Median (range)	42.4 (27.1-55.7)		46.9 (19.0-63.1)		—^b^		—		—	
	**Physical component subscale (n=47)**											
		Mean (SD)	50.7 (6.61)		52.3 (6.37)		3.1		.12		0.228 (small)	
		Median (range)	51.0 (35.8-62.6)		54.6 (37.3-59.6)		—		—		—	
**WEMWBS^c^ (n=47)**												
	Mean (SD)		48.3 (8.56)		51.7 (8.41)		7.0		.002		0.484 (small)	
	Median (range)		50.0 (26.0-67.0)		53.0 (33.0-64.0)		—		—		—	
**CES-D^d^ (n=45)**												
	Mean (SD)		15.5 (10.8)		11.5 (12.2)		–25.8		.02		0.349 (small)	
	Median (range)		13.0 (0-50.0)		6.00 (0-46.0)		—		—		—	
**FACIT-F^e^ (n=44)**												
	Mean (SD)		37.1 (8.73)		39.5 (8.71)		6.5		.048		0.306 (small)	
	Median (range)		39.0 (14.0-51.0)		42.0 (19.0-51.0)		—		—		—	
**ISI^f^ (n=44)**												
	Mean (SD)		9.67 (5.39)		8.42 (5.79)		–12.9		.08		0.265 (small)	
	Median (range)		10.0 (0-24.0)		8.00 (0-22.0)		—		—		—	

^a^SF-12: 12-item Short Form Health Survey (version 2).

^b^Not applicable.

^c^WEMWBS: Warwick-Edinburgh Mental Well-Being Scale.

^d^CES-D: Center for Epidemiologic Studies Depression Scale.

^e^FACIT-F: Functional Assessment of Chronic Illness Therapy–Fatigue.

^f^ISI: Insomnia Severity Index.

### Physical Capabilities

There was no time×group interaction for any physical quality, but a significant increase was found for flexibility (*P*=.049; Cohen *d*=0.233), isometric force of the lower limb (*P*=.002, Cohen *d*=0.381), estimated VO_2_max (*P*<.001; Cohen *d*=0.404), reaction time (*P*<.001; Cohen *d*=0.705), power output (*P*=.003; Cohen *d*=0.355), and balance (both for the left and right leg, *P*=.003; Cohen *d*=0.359 and *P*=.048; Cohen *d*=0.234, respectively). Surprisingly, a significant decrease was found on handgrip strength (*P*<.001; Cohen *d*=0.537; Table 4).

At posttest, of the 74 participants, 1 (1%) participant did not complete the Astrand test. The power output was set 50 W higher than that at pretest because the participant’s heart rate did not increase during the first minutes. For the balance test, 3 (4%) participants fell at pretest (only for 1 side) and 3 (4%) participants fell at posttest (only for 1 side). In order to be able to analyze the data, the maximal value of the whole data set for each fall had been inputted. For the leg extension, 1 (1%) participant did not complete the test at posttest due to back pain. For the force-velocity profile, Pmax was associated with an *r*^2^ value of 0.871 and 0.896 at the pretest and posttest time points, respectively.

**Table 4:** Changes in physical capabilities at pre- and postintervention time points.

			Pretest (n=74)	Posttest (n=74)	Change (%)	*P* value	Effect size
**Body mass (kg)**							
	Mean (SD)		76.4 (17.1)	76.2 (16.8)	–0.3	.54	0.071 (negligible)
	Median (range)		74.3 (49.4-136)	73.9 (50.2-136)	—^a^	—	—
**Body fat (%)**							
	Mean (SD)		26.3 (9.03)	27.1 (9.29)	3	.13	0.178 (negligible)
	Median (range)		26.4 (10.3-50.6)	27.0 (8.70-52.4)	—	—	—
**Flexibility (cm)**							
	Mean (SD)		25.9 (9.45)	26.9 (8.53)	3.9	.049	0.233 (small)
	Median (range)		26.7 (3.40-45.2)	26.7 (11.7-46.6)	—	—	—
**Balance**							
	**Ellipse area left (mm²)**						
		Mean (SD)	215 (194)	163 (138)	–24.2	.003	0.359 (small)
		Median (range)	151 (25.7-886)	110 (19.1-886)	—	—	—
	**Ellipse area right (mm²)**						
		Mean (SD)	186 (137)	162 (156)	–12.9	.048	0.234 (small)
		Median (range)	152 (40.7-795)	115 (29.4-795)	—	—	—
**Reaction time (ms)**							
	Mean (SD)		0.680 (0.0630)	0.633 (0.0788)	–6.9	<.001	0.705 (moderate)
	Median (range)		0.680 (0.573-0.861)	0.636 (0.416-0.842)	—	—	—
**Handgrip strength (kg)**							
	Mean (SD)		68.6 (16.7)	65.7 (16.9)	–4.2	<.001	0.537 (moderate)
	Median (range)		62.3 (41.1-108)	60.6 (37.0-106)	—	—	—
**Estimated VO**_**2**_ **max (mL/min/kg; n=73)**							
	Mean (SD)		26.8 (9.48)	29.0 (10.1)	8.2	<.001	0.404 (small)
	Median (range)		24.9 (7.06-53.7)	28.4 (7.34-54.6)	—	—	—
**Pmax (W/kg)**							
	Mean (SD)		7.63 (2.11)	7.82 (2.14)	2.5	.003	0.355 (small)
	Median (range)		7.02 (3.01-13.0)	7.35 (2.88-13.4)	—	—	—
**Lower limb isometric force (N m; n=73)**							
	Mean (SD)		328 (109)	347 (121)	5.8	.002	0.381 (small)
	Median (range)		308 (143-614)	329 (158-725)	—	—	—

^a^Not applicable.

### Link Between Variables

No correlations were found between the pretest-posttest changes in the objectives measures (ie, physical capabilities) and the subjective outcomes (ie, questionnaires). There was a strong correlation between changes in CES-D and WEMWBS scores (*r*=–0.76; *P*<.001). Correlations were also found between changes in CES-D and SF-12 scores (MCS; *r*=–0.64; *P*<.001), CES-D and ISI scores (*r*=0.57; *P*<.001), CES-D and FACIT-F scores (*r*=–0.58; *P*<.001), SF-12 and WEMWBS scores (*r*=0.67; *P*<.001), and FACIT-F and ISI scores (*r*=–0.59; *P*<.001).

## Discussion

### Principal Findings

This study was conducted to evaluate the impact of a personalized intervention, comprising an initial physical assessment and a supportive mobile app, on self-reported outcomes such as quality of life and objective physical capacities measurements. The key findings reveal that although the overall quality of life and the physical capacities significantly increased during the training period when the 2 groups were combined, no group effect was observed, meaning the PT group did not improve subjective and objectives measures significantly more than the FT group. This lack of significant difference between groups may be attributed to the limited engagement with the PT program.

### Duration, Intensity, and Workload of the Training Interventions

A significant increase in PA duration (*P*<.001), intensity (*P*<.001), and total workload (*P*<.001) was observed during the 4 months of the study. We recruited participants who self-declared being poorly active (ie, <1 hour of PA/wk). However, 18.2 minutes of PA per day were reported at pretest, that is, 127 minutes per week, which is more than what was expected, although still 23 minutes below the cutoff between active and inactive. Several reasons can explain this result. First, the participants were asked to manually add everything they were doing (walking, cleaning the house, gardening, etc), and they were probably initially not considering this type of activity as PA. Second, a large variability of PA was observed (SD 14.8 min/d), meaning that many participants matched our criteria perfectly, whereas others did not. In order to determine if this relatively high volume of PA at the pretest time point had an impact in our analysis, we performed the same analysis (mixed model, ie, 2-way ANOVA pretest-posttest×PT/FT) without participants reporting >150 minutes of PA per week at the pretest time point. The main conclusions were the same; that is, a time effect was found, but there was no time×group interaction. Finally, what could have led to this relatively high volume of PA at the pretest time point is the Hawthorne effect [38] since the participants self-reported their PA, making them potentially subject to social desirability [39]. In other words, because the participants were observed, they may have manually added more PA than what they were doing. This effect is unlikely to have an effect on the difference of PA between the pretest and posttest time points, but the volume of PA itself may have been impacted.

The difference between pretest and posttest measures could actually have been even higher. In fact, some participants mentioned that after reaching the goal of 150 minutes of PA per week, they stopped entering their PA in the mobile app, despite the guidelines. As a result, the true difference between pretest and posttest in terms of PA duration may have been artificially reduced. In addition, we observed a decrease of house-cleaning time between pretest and posttest, which is probably because people considered this activity as PA at pretest but no longer included it at posttest, which is in line with our results showing a significant increase in PA intensity (*P*<.001). Therefore, we can speculate that if 100% of PA had been manually entered in the mobile app, the difference between pretest and posttest measures would have been greater.

We did not observe any time×group effect for the PA duration, which was not totally unexpected since we asked both groups to complete at least 150 minutes of PA each week. However, a slightly greater volume in the PT group was anticipated since they had PT, which is more motivating as noted by Jirathananuwat and Pongpirul [40] in their systematic meta-review about promoting PA in the workplace. In summary, both the total duration of PA and the intensity of exercise were significantly greater at posttest, which obviously led to a significant increase in the total workload (*P*<.001).

A 33.9% (38/112) dropout was observed in this study (74 participants came at posttest vs 112 at pretest), which is in accordance with the literature. For instance, a 37% dropout was observed in a pilot study using smartphone technology to reach 150 minutes of PA per week with the goal of losing weight in a program on obesity [41]. Genin et al [42] observed a 29% dropout between pretest and posttest (5 months) for an onsite PA program where a coach was coming in the company to coach workers 2 times a week. Dalgas et al [43] asked tertiary employees to exercise 5 times per week (10-minute sessions) for 10 weeks and observed a dropout rate of 22%.

### Questionnaires

In this study, the mental component of quality of life (MCS of SF-12) was improved after 4 months of training in both groups. The benefits of PA on mental health have been demonstrated in many papers (eg, the work by Kandola et al [44] and Dinas et al [45]), which matches the findings of this study that also showed a significant (*P*<.02) decrease in depression levels (CES-D). A significant increase in well-being (WEMWBS; *P*<.002) was also found following 4 months of a more active lifestyle, which is in accordance with the literature as well [46]. Similarly, an inverse relationship between fatigue and the level of PA has been reported in the general population [47,48], and it has been shown that a PA intervention is the best method to reduce fatigue in patients with chronic fatigue [43]. Again, our FACIT-F findings are in line with these results.

It has been shown that PA can improve overall sleep quality [10], a result that was not observed in this work. Although validated, the use of self-reported questionnaires to assess sleep quality (ISI), as well as the relatively low increase in the level of PA, may explain the lack of a beneficial effect of PA on sleep quality (*P*=.08). It is important to mention that this study took place between 2021 and 2022, that is, during the COVID-19 pandemic. In this work, low values of the mental component of the SF-12 have been reported, although a significant (*P*<.006) increase from pretest to posttest was observed. Those low values are in line with the decrease of the quality of life in the general population [49-51] due to the COVID-19 pandemic. In France, the National Institute of Statistics has published data showing that, in 2021, the quality of life of the general population was at its lowest for the last decade [52]. Finally, we did not observe an (*P*=.12) increase in the PCS of the SF-12, despite increases in the individual physical qualities themselves (see the subsequent section).

### Physical Capabilities

The slight increase in the level of PA was enough to induce improvement in most physical capabilities in the inactive population involved in this study. An 8.3% increase of VO_2_max, evaluated with a valid and reliable method [53], was found between pretest and posttest measures. This in line with the literature [54-56], although this increase is generally associated with high-intensity interval training [57] or polarized training [58] with high volumes of aerobic exercise. This work shows that for inactive people with low VO_2_max values, this important physiological outcome can be increased by being slightly more active. All but one of the other physical qualities were significantly increased between pretest and posttest time points. This finding was previously found in a study on tertiary employees who practice 2 PA sessions per week for a period of 5 months, thereby significantly increasing their overall level of fitness (cardiovascular, upper and lower body strength, and balance) [59]. Strength training appears to offer aging adults many physiological benefits such as increased muscle mass [60]. Increased strength has been shown to help individuals maintain their daily functioning and mobility as they age [61], suggesting that strength training may positively affect quality of life. Moreover, muscular weakness associates with lack of balance (in this study, the balance score improved by 10.9%), which correlates with an increased risk of falls [62,63]. In addition, flexibility increased by 3.9% in this study, whereas lack of flexibility is associated with a higher incidence of muscular injury, and greater flexibility favors a decrease in back pain [64].

The only objective outcome that was not positively associated with the training intervention was grip strength, which decreased from pretest to posttest. This result can be explained by the methodological issues related to the handgrip size. In fact, it has been shown in the literature that handgrip size could lead to different results [65] explained by the force-length relationship [66]. In this study, a custom handgrip was used, including the possibility to change handgrip size. An error was made with the settings of the handgrip between pretest (handgrip size to 4.8 cm) and posttest (handgrip size to 5.2 cm). In addition, no decrease in body weight or body fat was observed in this study, which is in accordance with the study by Swift et al [67]. The authors explained that weight loss is associated with a large volume of aerobic exercise, which is not what was asked to the participants in this study.

### Limitations

A limitation of our study was the management of automatic reminders and the collection of questionnaire data. Despite our efforts to systematize the process and allow participants to complete questionnaires autonomously, we observed variable response rates, with 44 (59%) to 47 (64%) of 74 participants filling out the questionnaires at the posttest time point (<20 days before or after the second evaluation), depending on the questionnaire. This variability was partly due to some participants forgetting to complete one of the questionnaires. Given the fully autonomous design of the study, we were not allowed to directly remind them to fill out the missing questionnaires. In addition, the automatic reminder system was based on the participant’s last response, meaning that any delay in completing the questionnaires resulted in a subsequent delay in sending follow-up reminders. For the posttest evaluations, we selected the questionnaire that was completed closest to the evaluation day, arbitrarily setting a cutoff of 20 days. We recommend future researchers conducting fully automated studies to set specific dates for questionnaire completion to minimize accumulated delays and to consider conducting the posttest questionnaire on the day of evaluation to ensure more consistent and reliable data collection. A second limitation is that the PA level was self-reported. Actigraphy could have led to a more objective assessment of the PA level; however, it was not possible due to the number of participants enrolled. A third limitation is the low use of the virtual coach for the PT group. Participants were asked to get at least 150 minutes of PA each week, whereas the use of the virtual coach was recommended but not mandatory. It appears that lot of participants were highly motivated to be enrolled in this study, but most of the participants of the PT group took part in some personal PA, on their own or by going to some clubs, and none used the virtual coach a lot. This may be partly due to the way exercises were presented in the mobile app. More work is needed to determine whether the PT program can lead to more optimized beneficial effects than a traditional one. For instance, a better gamification in the app could be proposed, and the way the mobile app adjusts the intervention to the weakness of the participant must be improved.

### Conclusions

To the best of our knowledge, this study is unprecedented in its provision of a fully automated, web-based program that is tailored to individuals based on their initial physical assessments. The low use of PT in the PT group likely explains why the participants did not improve subjective and objectives measures significantly more than those in the FT group. Enhancements to the virtual coach’s appeal are necessary before a definitive conclusion about PT can be made. Notably, participants expressed satisfaction with their study involvement, anticipated to foster greater motivation and consistency in their daily activities, an insight derived from a poststudy survey with a 60% (44/74) response rate. Ultimately, a small (ie, 6 min/d) but significant increase of daily PA was observed in this study, regardless of the type of intervention, that is, PT or FT. A slight increase in exercise intensity was also found, leading to an overall increase in the workload. These slight changes were enough to improve quality of life and well-being and to decrease fatigue and depression level in this sedentary or poorly active population. Moreover, improvements in physical capacities across the board underscore the notion that even slight increases in PA can profoundly influence overall fitness.

## Appendix

Multimedia Appendix 1 CONSORT-eHEALTH (V 1.6.1).

## Abbreviations

CES-D: Center for Epidemiologic Studies Depression Scale
FACIT-F: Functional Assessment of Chronic Illness Therapy–Fatigue
FT: free training
ISI: Insomnia Severity Index
MCS: mental component subscale
PA: physical activity
PT: personalized training
REDCap: Research Electronic Data Capture
RPE: rating of perceived exertion
SF-12: 12-item Short Form Health Survey
WEMWBS: Warwick-Edinburgh Mental Well-Being Scale
WHO: World Health Organization

## Data Availability

The data sets generated and analyzed during this study are available from the corresponding author on reasonable request.
